# Downregulation of adipose triglyceride lipase by EB viral‐encoded LMP2A links lipid accumulation to increased migration in nasopharyngeal carcinoma

**DOI:** 10.1002/1878-0261.12824

**Published:** 2020-11-08

**Authors:** Shixing Zheng, Liudmila Matskova, Xiaoying Zhou, Xue Xiao, Guangwu Huang, Zhe Zhang, Ingemar Ernberg

**Affiliations:** ^1^ Department of Microbiology, Tumor and Cell Biology Karolinska Institutet Stockholm Sweden; ^2^ Department of Otolaryngology‐Head & Neck Surgery First Affiliated Hospital of Guangxi Medical University Nanning China; ^3^ The School of Life Sciences Baltic Federal University Kaliningrad Russia; ^4^ Scientific Research Center Life Science Institute Guangxi Medical University Nanning China

**Keywords:** adipose triglycerol lipase, EBV, latent membrane protein 2A, lipid metabolism, migration, nasopharyngeal carcinoma

## Abstract

Epstein–Barr virus (EBV)‐associated nasopharyngeal carcinoma (NPC) is one of the most common human cancers in South‐East Asia exhibiting typical features of lipid accumulation. EBV‐encoded latent membrane protein 2A (LMP2A) is expressed in most NPCs enhancing migration and invasion. We recently showed an increased accumulation of lipid droplets in NPC, compared with normal nasopharyngeal epithelium. It is important to uncover the mechanism behind this lipid metabolic shift to better understand the pathogenesis of NPC and provide potential therapeutic targets. We show that LMP2A increased lipid accumulation in NPC cells. LMP2A could block lipid degradation by downregulating the lipolytic gene adipose triglycerol lipase (ATGL). This is in contrast to lipid accumulation due to enhanced lipid biosynthesis seen in many cancers. Suppression of ATGL resulted in enhanced migration *in vitro*, and ATGL was found downregulated in NPC biopsies. The reduced expression level of ATGL correlated with poor overall survival in NPC patients. Our findings reveal a new role of LMP2A in lipid metabolism, correlating with NPC patient survival depending on ATGL downregulation.

## Graphical abstract

Epstein–Barr virus (EBV)‐associated nasopharyngeal carcinoma (NPC) cells display conspicuous lipid accumulation. Here, we show that EBV‐encoded membrane protein 2A (LMP2A) induces lipid accumulation in NPC cells by inhibition of lipolytic gene, adipocyte triacylglycerol lipase (ATGL). Inhibition of ATGL results in enhanced lipid accumulation and migratory capacity in NPC cells. Loss of ATGL in NPC cells correlates with poor overall survival.

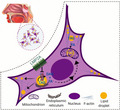

**Abbreviations**
ATGL: adipose triglycerol lipase
EBV: Epstein–Barr virus
ECAR: extracellular acidification rate
EIF4E: eukaryotic translation initiation factor 4E gene
FASN: fatty acid synthase
FCCP: carbonyl cyanide 4‐(trifluoromethoxy) phenylhydrazone
HSL: hormone‐sensitive lipase
IHC: immunohistochemistry
LC‐MC: liquid chromatography–mass spectrometry
LMP2A: latent membrane protein 2A
MGLL: monoglycerol lipase
NNE: normal nasopharyngeal epithelium
NPC: nasopharyngeal carcinoma
OCR: oxygen consumption rate
PEDF: pigment epithelium‐derived factor
PLS‐DA: partial least squares discriminant analysis
ROS: reactive oxygen species
siRNA: small interfering RNA

## 1 Introduction

Reprogrammed metabolism has been firmly established as a hallmark of cancer [1, 2]. Cancer is characterized by enhanced aerobic glycolysis [3, 4] and increased glutamine utilization [5]. Deregulated lipid metabolism is increasingly recognized and frequently reported in cancers [6, 7]. It is well recognized that lipids play diverse roles in maintaining cellular structure, forming membrane microdomains for functional scaffolding of protein complexes, serving as fat storage deposits, and acting as signaling molecules [8, 9]. Changes in lipid metabolism can affect numerous cellular processes, including cell motility, proliferation, differentiation, and growth [6, 7, 10]. Clinical studies also report that lipid metabolism‐related genes are correlated with prognosis in cancer patients. For example, fatty acid synthase (FASN) is upregulated in many cancers and high level of FASN expression is associated with poor prognosis in ovarian cancer [11], prostate cancer [12], lung cancer [13], malignant melanoma [14], and sarcomas [15].

Metabolic reprogramming is also a common feature of viral oncogenesis [16, 17]. As intracellular parasites, oncogenic viruses not only usurp the host's metabolic resources to meet the material and energy demands of their life cycles [18, 19] but also actively alter host cell metabolism by activating multiple cell signaling cascades which can contribute to cancer development [20]. Although rewired metabolism is a common feature in a variety of cancers, different cancers display different metabolic phenotypes with unique features. Revealing the underlying mechanism of the metabolic shift is important to uncover the pathogenesis of cancer and to provide potential therapeutic windows for targeting cancer through its metabolism.

Epstein–Barr virus (EBV) is a human oncovirus [21, 22]. It is estimated that EBV is associated with more than 200 000 new cases of cancer each year accounting for 1.8% of the global cancer burden, including nasopharyngeal carcinoma (NPC), Burkitt's lymphoma, a subset of Hodgkin's lymphoma, and gastric carcinomas [21, 23, 24]. Of note, a majority of the undifferentiated type of NPC (WHO type III) is EBV‐positive and this type is particularly common in NPC high incidence areas [25, 26, 27]. Latent infection with EBV is recognized as a factor in the pathogenesis of NPC [28]. During latent infection, EBV‐encoded latent membrane proteins 1 and 2A (LMP1 and LMP2A) play well‐documented roles in the process of NPC carcinogenesis [21, 29, 30, 31].

Like most cancers, NPC displays enhanced aerobic glycolysis and lipid accumulation as well. LMP1 plays a crucial role in enhanced aerobic glycolysis by activating multiple cell signaling cascades to alter the expression of various glycolytic genes, such as glucose transporter 1 [32, 33], hexokinase 2 [34], and l‐lactate dehydrogenase A chain [33]. Recently, we found a larger accumulation of lipid droplets in NPC tumor tissue as compared to the normal nasopharyngeal epithelium [35]. Adipocyte triglycerol lipase (ATGL or PNPLA2), a lipolytic gene, was significantly downregulated in NPC cell lines relative to normal nasopharyngeal epithelial cell lines. ATGL is the rate‐limiting enzyme for mobilizing triglycerol from lipid droplets. This mechanism of lipid accumulation in NPC needs to be further studied.

Based on metabolomic, transcriptomic, and biochemical data, we investigated the enhanced lipid accumulation in LMP2A‐expressing NPC cells. LMP2A showed a more significant effect on lipid metabolism than on glycolysis and glutamine metabolism. Interestingly, LMP2A‐associated lipid accumulation in NPC cells appears to depend on decreased catabolism of lipids due to lack of ATGL rather than on increased glycolysis or lipogenesis. Moreover, the increased lipid load correlated with the enhanced migratory properties previously observed in LMP2A‐positive NPC cells. Migration could be further enhanced by blocking ATGL expression. The degree of downregulation of ATGL in NPC correlates with poor overall survival. Our findings demonstrate a new role of LMP2A in lipid metabolism which also correlated with the overall survival of NPC patients depending on the downregulation of ATGL.

## 2 Materials and methods

### 2.1 Cell culture

Cell lines CNE1 (sex: female) [36] and TW03 (sex: male) [37] were originally established as derived from human nasopharyngeal carcinoma (NPC) cells. Parental NPC cells and LMP2A‐positive CNE1 and TW03 were cultured in modified Eagle's medium/high‐glucose medium (SH30243; HyClone, Logan, UT, USA) supplemented with 10% fetal bovine serum (10270098; Gibco, New York, USA) in the presence of streptomycin and penicillin (SV30010; HyClone). LMP2A‐positive cell lines were established by retroviral transduction as previously described [38, 39]. All cells were incubated at 37 °C in a humidified atmosphere containing 5% CO_2_.

### 2.2 Human specimens

The normal nasopharyngeal epithelium (NNE) from patients with no NPC and NPC biopsies was obtained from the Department of Otolaryngology‐Head & Neck Surgery, First Affiliated Hospital of Guangxi Medical University (Guangxi, China). Informed consent was obtained from each patient, and the tissue specimen collection was approved by the Research Ethics Committee of First Affiliated Hospital of Guangxi Medical University (Ref. No.: 2016‐175) and Ethics Committee of Karolinska Institutet (Ref. No.: 00‐312). The tissue array containing 132 cases of NPC with the clinical information for each case was purchased from Outdo Biotech (HNasN132Su01, Shanghai, China). The study methodologies conformed to the standards set by the Declaration of Helsinki.

### 2.3 *In vitro* small interfering RNA transfection

Cells were plated in a 10‐cm dish at an appropriate density 1 day before the transfection experiment to obtain a 30–50% confluent monolayer of cells on the day of transfection. Following the manufacturer's protocol, 60 pmol predesigned ATGL small interfering RNA (siRNA) (AM16708, ID#121867; Ambion, Austin, TX, USA) or siRNA control (AM4390844; Ambion) and 20 μL Lipofectamine RNAiMAX Reagent (13778; Invitrogen, Carlsbad, CA, USA) were diluted in 300 μL Opti‐MEM reduced serum medium, separately, to prepare an siRNA–lipid complex. After 5 min of incubation at room temperature, the siRNA–lipid complex was added to the cells. The transfection mix was removed after 24 h. Incubation and cells were incubated for 48 h when wound‐healing assay and BODIPY staining were performed.

### 2.4 RNA extraction, reverse transcription, and quantitative real‐time PCR

RNA was purified using the RNeasy Mini Kit (74106; Qiagen, Hilden, Germany), and cDNA was synthesized using Revert Aid First‐Strand cDNA Synthesis Kit (K1622, Fermentas; Thermo Fisher Scientific, Waltham, MA, USA) according to the manufacturer's protocols. qPCR was performed using the StepOnePlus Instrument (Applied Biosystems, Foster City, CA, USA) with a two‐step PCR amplification using SYBR Green (A25742; Applied Biosystems). Expression levels were normalized to the housekeeping eukaryotic translation initiation factor 4E gene (EIF4E). The primer sequences used for SYBR Green reactions were as follows: LMP2A—forward: 5′‐TGCAATTTGCCTAACATGGA‐3′ and LMP2A—reverse: 5′‐GAGCACAAGCATCACCAGGA‐3′; EIF4E—forward: 5′‐CATGGCTGATCCTGTCCTGAG‐3′ and EIF4E—reverse: 5′‐TAGGGGTGGTTTCCTGGGATT‐3′; ATGL—forward: 5′‐GGAGACCAAGTGGAACATCTCA‐3′ and ATGL—reverse: 5′‐AATAATGTTGGCACCTGCTTCA‐3′; HSL—forward: 5′‐CTCCTGCACAAATCCCGCTA‐3′ and HSL—reverse: 5′‐CGTCGCCCTCAAAGAAGAGT‐3′; and MGLL—forward: 5′‐CAACTGCTGAATGCCGTCTC‐3′ and MGLL—reverse: 5′‐AACTCCATGAGCAGGTAGGC‐3′. The PCR conditions were 95 °C for 30 s, followed by 40 cycles at 95 °C for 5 s and 60 °C for 30 s. Relative expression levels of target genes were determined by the 2^−ΔΔCt^ method. Each reaction was performed in triplicate.

### 2.5 Measurement of intracellular pyruvate, lactate, triglycerol, and acetyl‐CoA

Cells were seeded in 10‐cm culture dishes 1 day before all measurements. Ten million cells were collected and then resuspended and homogenized in 1 mL PBS. The cell debris was eliminated by centrifugation. The supernatant was deproteinized with perchloric acid using Deproteinizing Sample Preparation Kit (K808‐200; BioVison, Milpitas, CA, USA) before measurements. Intracellular pyruvate, lactate, and triglycerol levels were measured using Pyruvate Assay Kit, Lactic Acid Assay Kit, and Triglycerol Assay Kit (A081, A019, and A110, respectively; Nanjing Jiancheng Bioengineering Institute, Nanjing, China) at 505, 530, and 510 nm, respectively. Acetyl‐CoA concentration was measured using an Acetyl‐Coenzyme A Assay Kit (MAK039; Sigma‐Aldrich, St. Louis, MO, USA) according to the manufacturer's instructions (λex = 535/λem = 587 nm), and data were normalized to assay buffer. Dual‐Wave Violet Spectrophotometer (LAMBDA Bio 20, Waltham, MA, USA) was applied to measure the absorbance and fluorescence. Experiments were performed according to the manufacturer's instructions using three independent biological repeats.

### 2.6 Immunofluorescence assay and BODIPY (493/503) staining

As previously described [40], cells were cultured to attach and form a monolayer before staining. Cells were fixed in 4% paraformaldehyde solution for 10 min, permeabilized with 0.1% saponin for 30 min, and blocked with 5% BSA for 30 min. Anti‐ATGL antibody (sc‐365278, RRID: AB_10859044; Santa Cruz Biotechnology, Santa Cruz, CA, USA) was diluted 1 : 50 in PBS and incubated on slides in a humidified chamber at 4 °C overnight. Then, the slides were washed with PBS and incubated with 1 : 800 Alexa Fluor 594 (A‐21203; Invitrogen) for 1 h at room temperature. Lipid droplets were stained with BODIPY 493/503 (D3922; Life Technologies, Carlsbad, CA, USA) at final concentration of 5 μg·mL^−1^ for 30 min, and nuclei were stained with 1 μg·mL^−1^ Hoechst 33258 (Sigma‐Aldrich) for 10 min. Images were captured by an Apotome/Axiovert 200M Microscope (Carl Zeiss, Oberkochen, Germany) using an Apochromat 63X objective lens with a 1.40 numerical aperture.

### 2.7 Migration assay

Migration was evaluated using the ibidi Culture‐Insert (GmbH, Gräfelfing, Germany). The ibidi Culture‐Insert provides reservoirs for culturing cells that are separated by a 500‐μm‐thick wall. Due to the specially designed silicone bottom, the Culture‐Inserts stick to the surface, preventing any cell growth beneath the walls. Briefly, cells were seeded at 60 000 cells per reservoir, which would allow creating a confluent monolayer the day after. Cell‐free gaps were created onto confluent cells after removal of the insert. The plates were photographed to fix an initial width of the gaps. Then, cells were cultured in the presence or absence of 150 μm 1‐butanol (281549; Sigma‐Aldrich) or 30 μm atglistatin (SML1075; Sigma‐Aldrich). Atglistatin was originally discovered as an inhibitor of ATGL in mice, but has later been shown to also specifically inhibit human ATGL [41, 42, 43, 44, 45]. The widths of the gaps were evaluated again 8–15 h later. The closure of gaps was determined by comparing images at the beginning and endpoint using image j software (National Institutes of Health, Bethesda, MD, USA).

### 2.8 Microarray

Total RNA from LMP2A‐positive and LMP2A‐negative NPC cells was extracted with RNeasy Mini Kit (74106; Qiagen). One biological replicate of each sample was prepared. Gene expression microarray was carried out at GMINIX Informatics Limited Corporation (Shanghai, China) using Affymetrix Human Gene 1.0 Chip according to the standard protocol. Profiling of gene expression, Gene Ontology analysis, and KEGG pathway analysis were performed at the platform of gminix using r statistical software packages (R Foundation for Statistical Computing, Vienna, Austria).

### 2.9 Meta‐analysis of microarrays

Six publicly available datasets from Gene Expression Omnibus were included in the meta‐analysis. Raw data were normalized as described above. The ATGL expression in these six microarrays was pooled using revman 5.3 meta‐analysis software (The Cochrane Collaboration, Copenhagen, Denmark) applying the fixed‐effect inverse‐variance model. The standard mean difference was used to reflect the difference across microarrays. A funnel plot was constructed to evaluate data symmetry and publication bias. Forest plots were generated to represent the standard mean difference with the corresponding 95% confidence intervals across the included microarrays.

### 2.10 Metabolomics

Twenty‐four hours before cell collection, 2.0 × 10^6^ cells were seeded in 10‐cm culture dishes. Ten million cells of each sample were harvested at a nonconfluent density. Cell pellets collected by centrifugation at 1500 ***g*** for 5 min were washed with prechilled PBS twice and 0.9% w/v NaCl once. Metabolism of cells was arrested by freezing in liquid nitrogen.

Nontargeted liquid chromatography–mass spectrometry (LC‐MS) was performed by Applied Protein Technology Corporation (Shanghai, China) using Agilent 1290 Infinity LC System (Agilent, Waldbronn, Germany) and triple time‐of‐flight 5600+ mass spectrometer (AB SCIEX, Framingham, MA, USA). Briefly, cell pellets were resuspended in 1 mL of methanol/acetonitrile/water (2 : 2 : 1, v/v), proceeded further with low‐temperature ultrasound pyrolysis for 20 min, and centrifuged for 20 min at 14 000 ***g***. The supernatant was mixed with a 100 μL acetonitrile/water solution (1 : 1, v/v), centrifuged for 15 min at 14 000 ***g*** at 4 °C, and transferred to an LC‐MS vial with insert. Lipid extracts were separated by Waters ACQUITY UPLC BEH Amide 1.7 μm, 2.1 × 100 mm column (Waters Corporation, Milford, MA, USA) and Waters ACQUITY UPLC HSS T3 1.8 μm, 2.1 × 100 mm column (Waters Corporation), maintained at 25 °C. Triple time‐of‐flight 5600+ dual electrospray ionization mass spectrometer was used. Raw data were converted into the mzXML format by Proteo Wizard [46] and proceeded by xcms software [47, 48] for feature detection, retention time correction, and alignment. A fold change of > 1.2 was chosen for the variables in the projection. metaboanalyst 4.0 [49] was used to generate partial least squares discriminant analysis (PLS‐DA) and metabolic pathway analysis. Based on the KEGG metabolic pathways database, the software conducts pathway enrichment and topology analysis to identify pathways that are most significantly impacted under the specific experimental conditions.

### 2.11 Bioinformatics analysis

Integrated lipid metabolic pathway analysis on results obtained by combining the metabolomic and microarray data was performed by Joint pathway analysis module of metaboanalyst 4.0 [49].

### 2.12 Seahorse extracellular flux analysis

For Seahorse analysis, 50 000 cells were seeded in Agilent Seahorse 24‐well cell culture microplate and cultured in DMEM + 10% FBS 16 h at 37 °C in a humidified atmosphere containing 5% CO_2_ before the experiment. Sensor cartridges were hydrated in Seahorse XFe24 Calibrant at 37 °C in a non‐CO_2_ incubator overnight. Cellular oxygen consumption rate (OCR) and extracellular acidification rate (ECAR) were measured by Seahorse XFe24 extracellular flux analyzer (Agilent).

Cell metabolic phenotype was investigated according to the user guide for Seahorse XF Cell Energy Phenotype Test Kit. Before the run, culture media were replaced with pH‐adjusted (pH = 7.4 ± 0.5) Seahorse Base Media supplied with 10 mm glucose (G8270; Sigma‐Aldrich) and 1 mm sodium pyruvate (S9636; Sigma‐Aldrich). OCR and ECAR were simultaneously measured before and after injection of the mitochondrial stressor. The stressor mix contains oligomycin and carbonyl cyanide 4‐(trifluoromethoxy) phenylhydrazone (FCCP) at the final concentration of 1 and 0.5 μm, respectively.

Glycolysis levels were determined by monitoring ECAR at different conditions, according to the protocol of Seahorse XF glycolysis stress test. The Seahorse XF glycolysis stress test is a standard assay for measuring glycolytic function in cells by directly measuring the ECAR, as the schematic diagram shown in Fig. 3A. Culture media were replaced with pH‐adjusted adjusted (pH = 7.4 ± 0.5) Seahorse Base Media supplied with 1 mm l‐glutamine (G7513; Sigma‐Aldrich). To measure ECAR, glucose, oligomycin, and 2‐deoxy‐d‐glucose were sequentially injected at a final concentration of 10 mm, 1 μm, and 50 mm, respectively.

Mitochondrial function was evaluated as described in the Seahorse XF Mito Stress Test Kit user guide, as the schematic diagram shown in Fig. 3J. Culture media were replaced with the same media as used for cell metabolic phenotype test before the run. Basal levels of OCR were recorded, followed by OCR measurement after oligomycin, FCCP, and rotenone sequentially injected at the final concentration of 100, 0.5, and 0.5 μm, respectively.

Statistical analysis was conducted as described in the Report Generator user guide for each kit. Student's *t*‐test was applied to the comparison between LMP2A‐positive and LMP2A‐negative NPC cells.

### 2.13 Immunohistochemistry

The sections of NNE and NPC tissues were deparaffinized in xylene, hydrated in graded alcohol solutions, and proceeded for heat‐induced antigen retrieval in citrate buffer (0.01 m, pH 6.0) for 20 min at 95–100 °C. Nonspecific binding sites were blocked with serum‐free blocking reagent of EnVision Detection Systems (K500711‐2; Dako, Agilent Technologies, Inc., Santa Clara, CA, USA) for 30 min at room temperature. Specific anti‐ATGL (sc‐365278, RRID: AB_10859044; Santa Cruz Biotechnology) was applied overnight at 4 °C. Immunodetection was carried out with EnVision Detection Systems (K500711‐2; DAKO) for 30 min. 3,3‐Diaminobenzidine (K500711‐2; Dako) was then used to visualize nuclei. Finally, sections were counterstained with hematoxylin. For negative control, several NPC sections were incubated with isotype‐matched IgG instead of the primary antibody. Five images of the sections were acquired and scored blindly by two pathologists as follows: score = proportion of positive stain (0, < 10%; 1, 10–25%; 2, 25–50%; 3, 50–74%; 4, > 75%) × mean stain intensity (0–3). The total immunohistochemistry (IHC) scores were used to divide patients into two equal groups with either high or low ATGL expression. The Kaplan–Meier method was used to estimate overall survival, and the log‐rank test was used to evaluate differences between survival curves. Statistical analyses were performed using Student's *t*‐test or Welch's *t*‐test (normal distribution) by graphpad prism 6.0 (GraphPad Software, San Diego, CA, USA). A *P*‐value of < 0.05 was considered statistically significant.

### 2.14 *In vitro* DNA transfection for ATGL overexpression

Cells were plated in a 10‐cm dish at an appropriate density 1 day before the transfection experiment to get 30–50% confluent on the day of transfection. Following the manufacturer's protocol, 5 μg ATGL plasmid DNA was mixed with 15 μL of the FuGENE HD (E2311; Promega, Madison, WI, USA) in 500 μL Opti‐MEM reduced serum medium and then incubated for 10 min before adding to the dish of cells to be transfected. The transfection mix was removed after 24‐h incubation, and cells were incubated for 48 h before subsequent wound‐healing assay, BODIPY staining, and western blotting.

### 2.15 Immunoblotting

Protein lysates were prepared and analyzed by a conventional western blot (WB) assay as described elsewhere [39]. Signals from enhanced chemiluminescence reagent (ECL, Amersham, Piscataway, NJ, USA), used in the WB assay, were acquired by a ChemiDoc XRS + (Bio‐Rad Laboratories, Hercules, CA, USA) with image lab™ software (Bio‐Rad Laboratories, Hercules, CA, USA). Antibodies, anti‐ATGL (sc‐365278, RRID: AB_10859044; Santa Cruz Biotechnology) at 1 : 1000, actin (C4) : sc‐47778, and anti‐mouse IgG‐HRP (170‐6516), were obtained from Bio‐Rad Laboratories.

### 2.16 Detection of intracellular reactive oxygen species

Intracellular reactive oxygen species (ROS) production was measured using 2′,7′‐dichlorofluorescein diacetate (DCFH) (Cat #K936; BioVision, Milpitas, CA, USA), which is oxidized to the fluorescent product 2′,7′‐dichlorofluorescein (DCF) by ROS. Cells were seeded in 96‐well flat clear bottom black microplate overnight to obtain about 80% confluency. After washing cells three times with ROS assay buffer, 100 μL of 1× ROS label diluted in ROS assay buffer was added into each well and then cells were incubated for 45 min at 37 °C in the dark. ROS label was removed, and 100 μL ROS assay buffer was added to the wells. For fluorescence microscope analysis, cells were observed using Leica DMRE Laser Scanning Confocal Research Epi‐Fluorescence Microscope TCS SP2 (Wetzlar, Germany). To quantify the intracellular ROS, some amount of cells were seed in 96‐well plate and the fluorescence was measured at Ex/Em = 495/529 nm with Dual‐Wave Violet Spectrophotometer (LAMBDA Bio 20). Data were normalized to those obtained from cells incubated in serum‐free DMEM. Experiments were performed according to instructions using three independent biological repeats.

## 3 Results

### 3.1 LMP2A induces metabolic shift and rewires lipid metabolism pathway

**Fig. 1:**
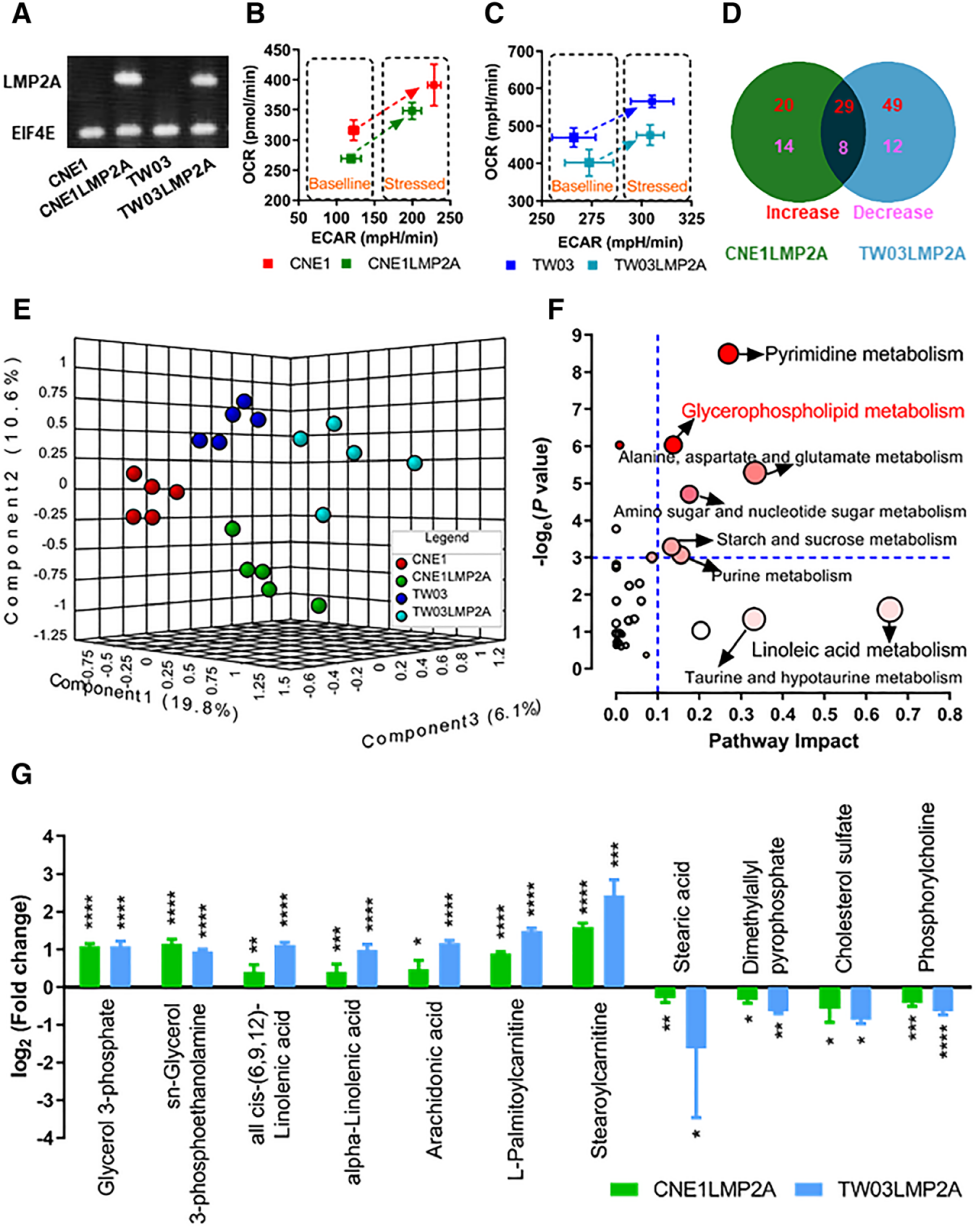
LMP2A‐positive NPC cell lines differ from parental cells in metabolic profile. (A) NPC cell lines express LMP2A. A representative gel electrophoresis analysis of RT–qPCR products (*n* = 2/group). (B, C) Metabolic phenotype analysis by simultaneously measuring the OCR and ECAR. (D) A two‐set Venn diagram showing differential metabolites between LMP2A‐positive and LMP2A‐negative NPC cell lines (CNE1 and TW03) by LC‐MS analysis. Indicated in the diagram are the numbers of upregulated and downregulated metabolites. (E) PLS‐DA 3D scores plot. (F) Metabolomic pathway analysis as generated by metaboanalyst software package. All the matched pathways are displayed as circles. The color of each circle is based on −log_e_ (*P*‐value) (darker colors indicate more significant changes in metabolites in the corresponding pathway), whereas the size of the circle corresponds to the pathway impact score. (G) List of differential lipid metabolites in LMP2A‐positive NPC cell lines. Data are presented as means ± SD; for B and C, *n* = 4/group; and for G, *n* = 5/group.

To explore the effect of LMP2A on cellular metabolism, we transfected an LMP2A‐expressing construct into two EBV‐negative human NPC‐derived cell lines (CNE1 and TW03) and established stable transfectants expressing LMP2A (Fig. 1A). The bioenergetic profiles of these cells were characterized using the Seahorse XFe analyzer. The mitochondrial function was evaluated by measuring OCR and glycolysis activity by ECAR due to lactate production under baseline and stressed conditions. The LMP2A‐positive cells showed a low OCR and high ECAR metabolic phenotype (Fig. 1B,C). A nontargeted metabolomic approach was applied to detect differences in metabolite production between LMP2A‐positive and LMP2A‐negative NPC cells at the cellular level. Forty‐nine metabolites increased and 22 decreased in CNE1LMP2A, while 78 increased and 20 decreased in TW03LMP2A, compared with the parental cells, respectively (*P* < 0.05 by *t*‐test using 1.2‐fold change as cutoff; Fig. 1D). Of these, twenty‐nine metabolites were increased in both of the LMP2A‐positive cell lines (Fig. 1D). The metabolic profiles of LMP2A‐positive cells were clearly discriminated from those of the negative control cells as shown by applying PLS‐DA (Fig. 1E). The pathway impact analysis was used to identify the metabolic pathways affected by LMP2A. The following five metabolic pathways were significantly related to LMP2A expression: pyrimidine metabolism; glycerophospholipid metabolism; alanine, aspartate, and glutamate metabolism; amino sugar; and nucleotide sugar metabolism and starch and sucrose metabolism (Fig. 1F). In particular, several classes of lipids showed significant differences between LMP2A‐positive and LMP2A‐negative cells (Fig. 1G).

### 3.2 Lipid accumulation confers enhanced migration in LMP2A‐positive cells

**Fig. 2:**
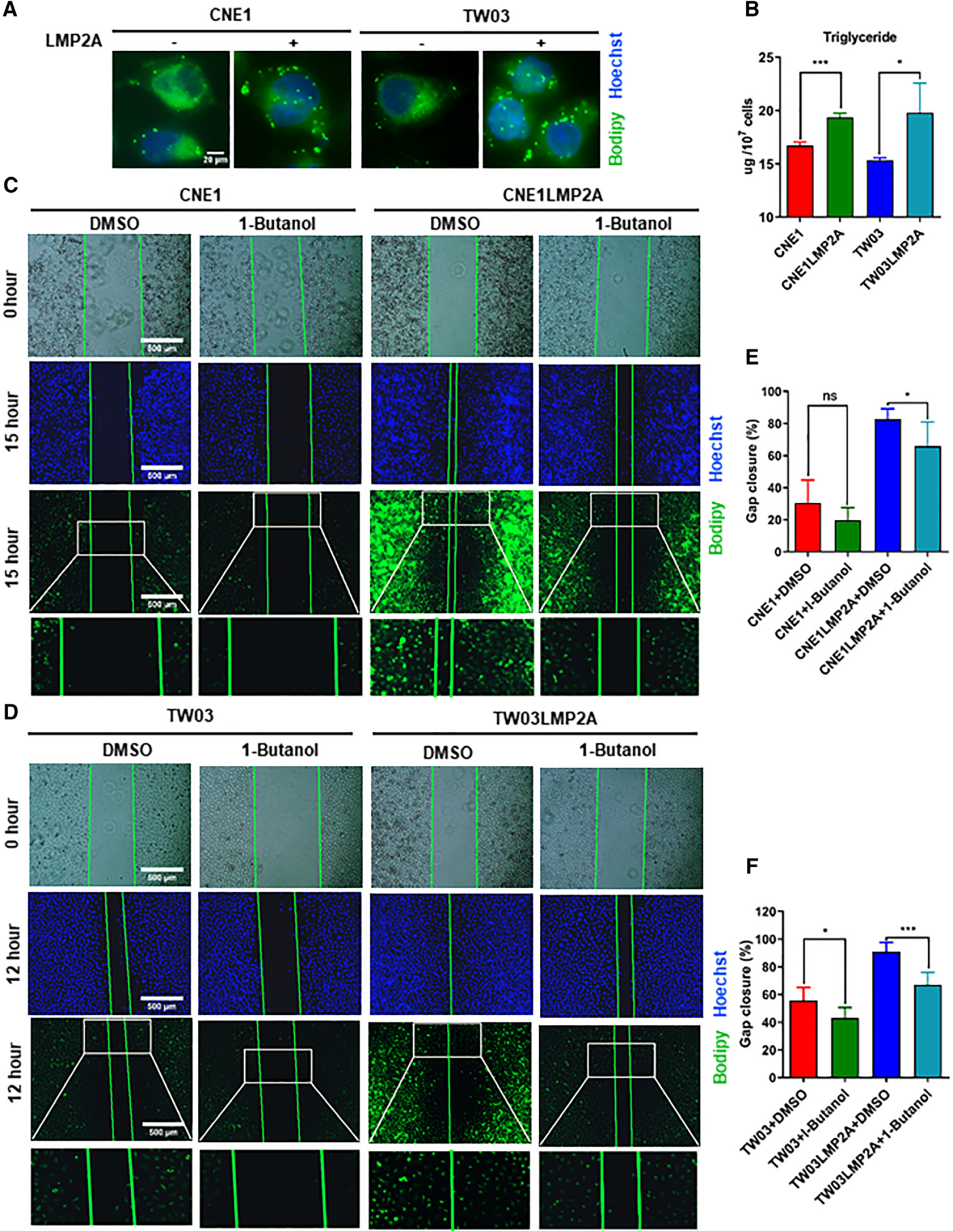
LMP2A induces lipid accumulation in parallel to cell migration capacity in NPC cell lines. (A) Staining of lipid droplets with BODIPY (493/503) (green) and nuclei with Hoechst (blue). Scale bar = 20 μm. (B) Intracellular triglycerol measurement with GPO‐PAP method. (C, D) Migration assay. Images were taken at two time points after creating a cell‐free zone in cells cultured in the presence of DMSO or 1‐butanol. Microscope analysis of fluorescence upon staining of lipid droplets with BODIPY (493/503) (green) and nuclei with Hoechst (blue) in the presence of DMSO or 1‐butanol (the lower two panels). Scale bar = 500 μm. (E, F) Gap closures were measured. Images reported in A, C, and D are representative of *n* = 3 independent experiments. Data are presented as means ± SD; for B, E, and F, *n* = 3/group. *P < 0.05 and ***P < 0.001 as determined by Student's *t*‐test.

We detected an increase in lipid droplets in LMP2A‐positive cells (Fig. 2A) also in line with our data obtained by LC‐MS (Fig. 1G). There were more triglycerols in LMP2A‐positive cells as compared to the negative cells (Fig. 2B).

The migration of LMP2A‐positive cells was affected by lipid metabolism, as shown by inhibiting phospholipase D with 1‐butanol. We performed a wound‐healing assay to evaluate the migratory ability of cells cultured with or without 1‐butanol. This treatment significantly decreased the lipids as shown by BODIPY staining at the endpoint (Fig. 2C,D, bottom two lines). The lipid‐depleted, 1‐butanol‐treated LMP2A‐positive cells demonstrated a significantly slower gap closure than untreated LMP2A‐positive cells. As expected, 1‐butanol treatment had less impact on the gap closure of LMP2A‐negative cells, as shown in Fig. 2C–F.

### 3.3 LMP2A‐induced lipid accumulation in NPC cells is independent of glucose consumption

**Fig. 3:**
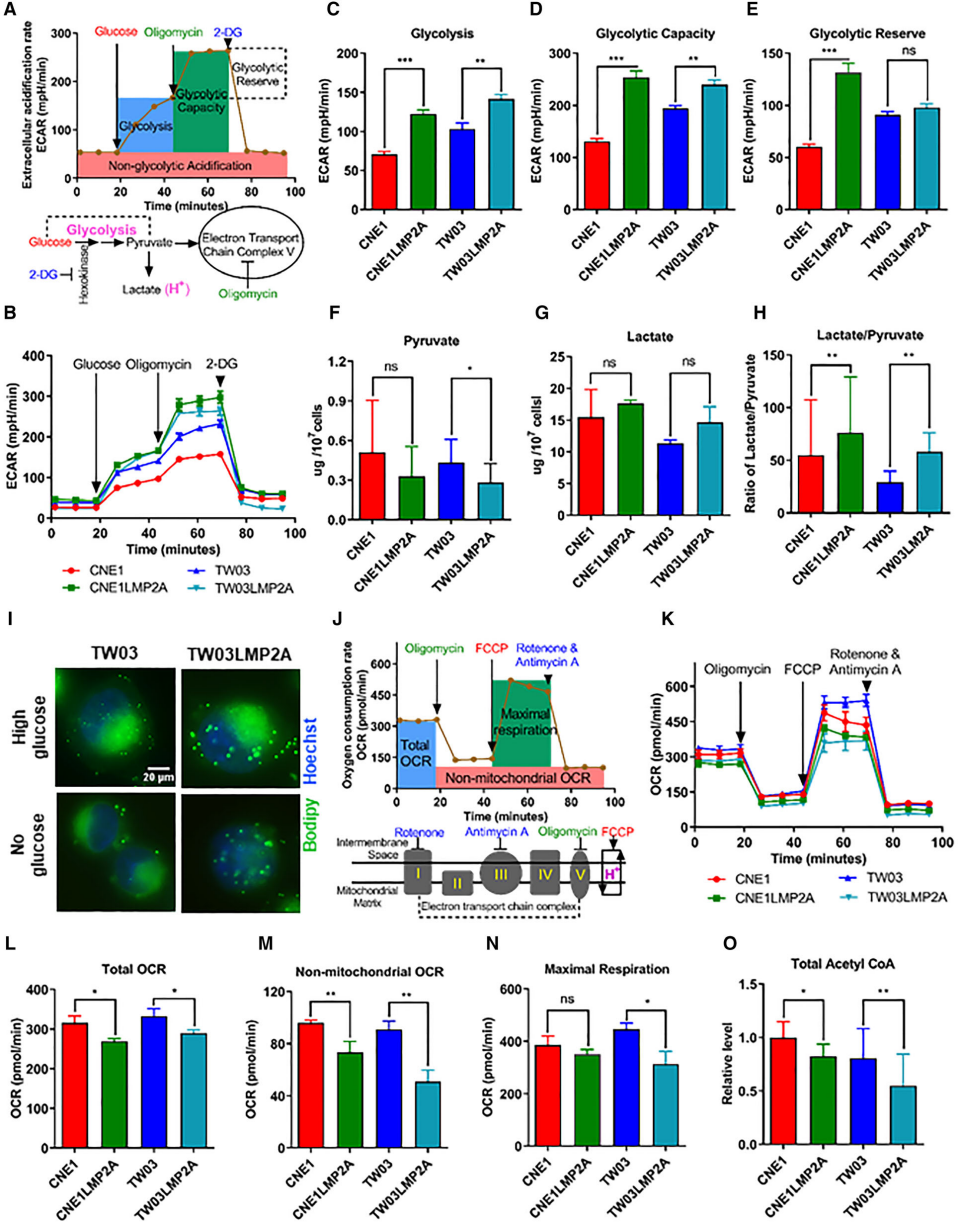
LMP2A‐positive cells showed enhanced glycolysis and decreased bioenergetic activities of mitochondria. (A) Schematic of examining the multiple parameters of glycolytic activity. Glucose fuels glycolysis; oligomycin inhibits ATP synthase in mitochondria; and 2‐DG is a competitive inhibitor of glucose intake. (B) ECAR in LMP2A‐positive and LMP2A‐negative NPC cell lines. (C–E) Glycolysis, glycolytic capacity, and glycolytic reserve based on ECAR profile (Panel B). (F, G) Colorimetry‐defined intracellular lactate and pyruvate levels. (H) The ratio of the intracellular lactate to pyruvate levels. (I) Lipid droplets were stained with BODIPY (493/503) (green) and nuclei with Hoechst (blue). Scale bar = 20 μm. (J) Schematic of examining the multiple parameters of oxidative phosphorylation activity. Oligomycin inhibits ATP synthase; FCCP uncouples oxygen consumption from ATP production; and rotenone and antimycin A block electron transport, enabling measurement of nonmitochondrial oxygen consumption. (K) OCR in LMP2A‐positive and LMP2A‐negative NPC cell lines measured by Seahorse XFe. (L–N) Total OCR, nonmitochondrial OCR, and maximal respiration based on OCR profile. (O) The relative amount of total acetyl‐CoA level as determined by a fluorescence microplate reader. Images reported in I are representative of *n* = 3 independent experiments. Data are presented as means ± SD; For B, C, D, E, F, H, K, L, M, N, and O, *n* = 3/group.*P < 0.05 and **P < 0.01 as determined by Student's *t*‐test.

We explored whether the increased lipid content of LMP2A‐positive NPC cells depended on glycolysis, comparing LMP2A‐positive and LMP2A‐negative cells using the Seahorse XFe glycolysis stress test (Fig. 3A,B). LMP2A‐positive NPC cells showed higher levels of glycolysis (Fig. 3C), glycolytic capacity (Fig. 3D) and glycolytic reserve (Fig. 3E). LMP2A expression did not significantly change the intracellular pyruvate (Fig. 3F) and lactate levels (Fig. 3G), but the ratio of lactate/pyruvate was significantly elevated in the LMP2A‐expressing cells (Fig. 3H). To investigate whether the increase in lipids in LMP2A NPC cells was the result of increased glycolysis, we cultured cells in media with or without glucose and compared the intracellular lipid load between LMP2A‐positive and LMP2A‐negative NPC cells. We used two different media compositions: DMEM with high glucose and a‐MEM without glucose. Although an increase in glucose in the culture medium facilitated lipid accumulation in the NPC cells, LMP2A‐positive cells contained more lipids as shown by BODIPY, even when cultured in glucose‐depleted media (Fig. 3I).

Since abnormal lipid accumulation in LMP2A‐positive cells could be due to deregulated mitochondrial function, we compared oxygen consumption between the LMP2A‐positive and LMP2A‐negative NPC cells using the Seahorse XFe Mito Stress Test (Fig. 3J,K). The LMP2A‐expressing cells showed a decreased total OCR at baseline conditions (Fig. 3L), at nonmitochondrial oxygen consumption (Fig. 3M) and during maximal respiration (Fig. 3N). To evaluate the biosynthetic activity of mitochondria, we measured intracellular acetyl‐coenzyme A and found that mitochondria in the LMP2A‐positive cells displayed lower biosynthetic activity of acetyl‐coenzyme A (Fig. 3O).

### 3.4 LMP2A affects the metabolic processing of lipids in NPC‐derived cell lines at the gene expression level

**Fig. 4:**
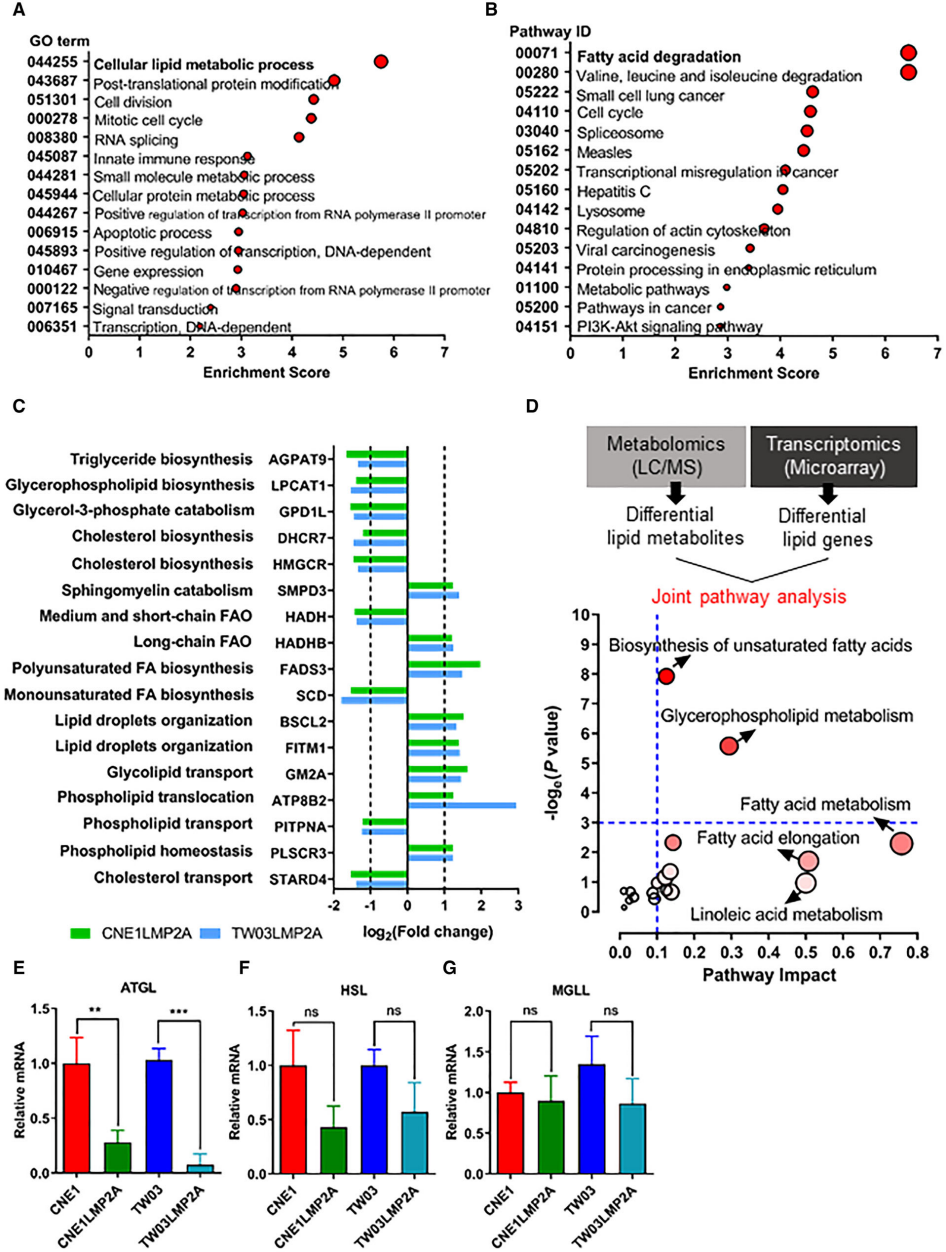
LMP2A affects the metabolic processing of lipids in NPC cell lines. (A, B) Top 15 GO terms and pathway IDs enriched, respectively, in A and B. The bar plot depicts the enrichment scores calculated by (nf/*n*)/(Nf/*N*). nf: the number of different genes in pathway; Nf: the number of different genes; *n*: the number of genes in pathway; and *N*: the total number of genes in the annotation system. *P* < 0.05 determined as difference by a Fisher exact test. (C) Analysis of microarray data reveals LMP2A affects lipid metabolism‐related genes at the mRNA level. (D) Integrated lipid metabolic pathway analysis of results obtained from combined metabolomic and microarray data. (E–G) RT–qPCR analysis of relative ATGL, HSL, and MGLL expression in LMP2A‐positive and LMP2A‐negative NPC cells, respectively. Data are presented as means ± SD; for A, B, and C, *n* = 1/group; and for E, F, and G, *n* = 3/group.**P < 0.01 and ***P < 0.001 as determined by Student's *t*‐test. AGPAT9, glycerol‐3‐phosphate acyltransferase 3; LPCAT1, lysophosphatidylcholine acyltransferase 1; GPD1L, glycerol‐3‐phosphate dehydrogenase 1‐like protein; DHCR7, 7‐dehydrocholesterol reductase; HMGCR, 3‐hydroxy‐3‐methylglutaryl‐coenzyme A reductase; SMPD3, sphingomyelin phosphodiesterase 3; HADH, hydroxyacyl‐coenzyme A dehydrogenase, mitochondrial; HADHB, trifunctional enzyme subunit beta, mitochondrial; FADS3, fatty acid desaturase 3; SCD, acyl‐CoA desaturase; BSCL2, Berardinelli–Seip congenital lipodystrophy type 2 protein; FITM1, fat storage‐inducing transmembrane protein 1; GM2A, ganglioside GM2 activator; ATP8B2, phospholipid‐transporting ATPase ID; PITPNA, phosphatidylinositol transfer protein alpha isoform; PLSCR3, phospholipid scramblase 3; STARD4, StAR‐related lipid transfer protein 4; ATGL, adipose triglycerol lipase; HSL, hormone‐sensitive lipase; MGLL, monoglycerol lipase.

To further elucidate the molecular mechanism of lipid accumulation in LMP2A‐positive cells, we screened differentially expressed genes involved in lipid metabolism by microarray. Expression of LMP2A resulted in changes in expression of transcripts involved in cellular lipid metabolism and in fatty acid degradation. These ranked high among the top 15 biological processes and top 15 biochemical pathways by gene ontology, respectively (Fig. 4A,B). Seventeen lipid metabolism‐related genes were identified as differentially expressed. Specifically, glycerol‐3‐phosphate acyltransferase 3 (AGPAT9), lysophosphatidylcholine acyltransferase 1 (LPCAT1), 7‐dehydrocholesterol reductase (DHCR7), and 3‐hydroxy‐3‐methylglutaryl‐coenzyme A reductase (HMGCR) are involved in lipid/cholesterol biosynthesis and were decreased in LMP2A‐positive cells. However, two genes involved in lipid/cholesterol catabolism were downregulated in LMP2A‐positive cells: glycerol‐3‐phosphate dehydrogenase 1‐like protein (GPD1L) and sphingomyelin phosphodiesterase 3 (SMPD3). A set of genes involved in lipid homeostasis, transport, or lipid storage were upregulated, such as Berardinelli–Seip congenital lipodystrophy type 2 protein (BSCL2), fat storage‐inducing transmembrane protein 1 (FITM1), ganglioside GM2 activator (GM2A), phospholipid‐transporting ATPase ID (ATP8B2), and phospholipid scramblase 3 (PLSCR3) (Fig. 4C). In order to map lipid metabolic pathways affected by LMP2A, we performed an integrated metabolic pathway analysis on the combined results obtained from metabolomic and transcriptomic analysis conducted under the same experimental conditions. Genes and metabolites involved in the biosynthesis of unsaturated fatty acids and in glycerophospholipid pathways showed differential expression and were enriched in LMP2A‐expressing cells (Fig. 4D). Three genes essential for lipid turnover were analyzed further. ATGL, hormone‐sensitive lipase (HSL), and monoglycerol lipase (MGLL) sequentially degrade triglycerols from lipid droplets. Their expression levels were validated by qPCR. ATGL mRNA was significantly decreased in LMP2A positive cells compared with parental cells, while the downregulation of mRNAs for HSL and MGLL genes was less conspicuous (Fig. 4E–G).

### 3.5 Inhibition of ATGL enhances cell migration capacity in NPC cell lines

**Fig. 5:**
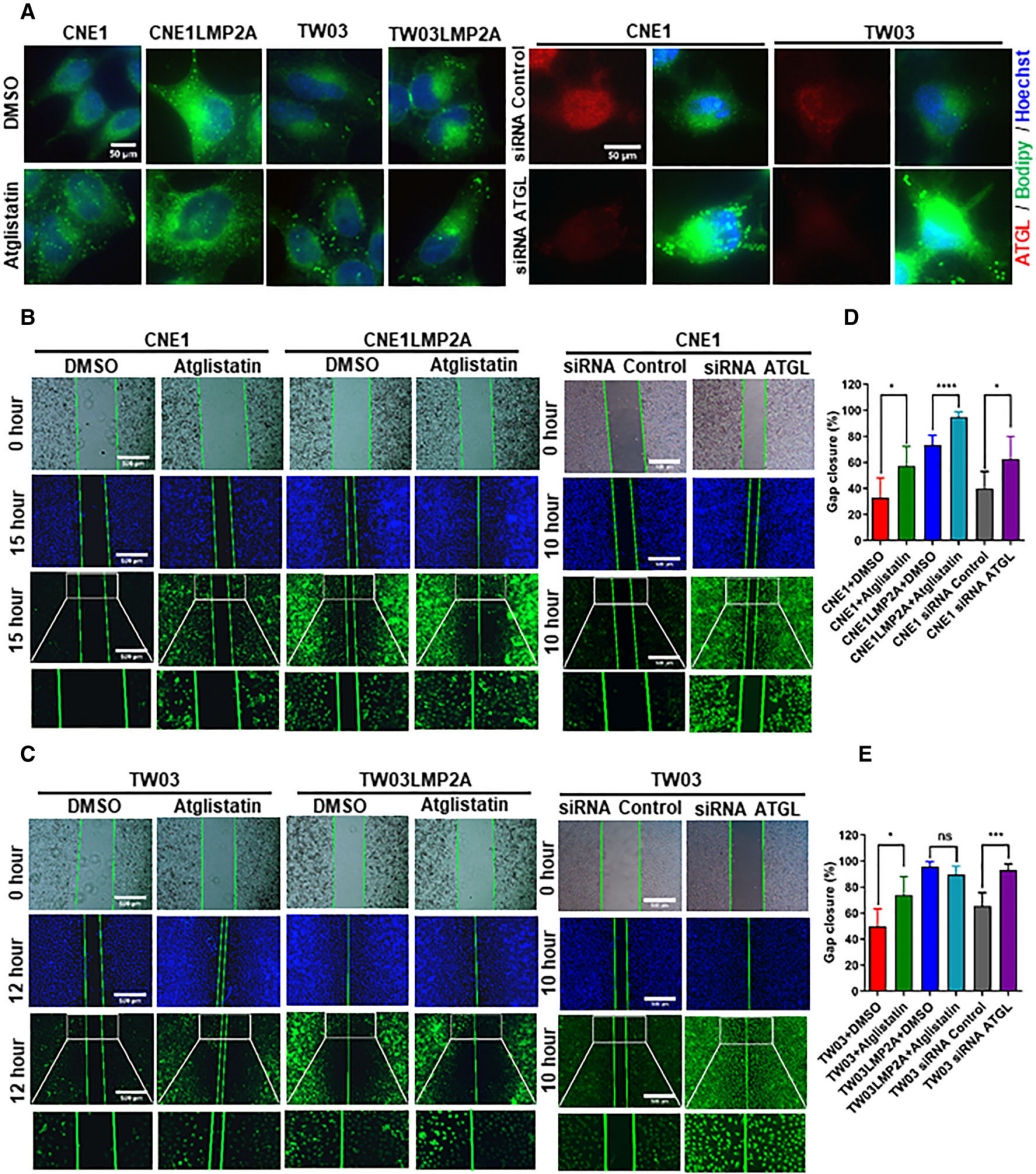
Inhibition of ATGL enhances cell migration capacity in NPC cell lines. (A) Staining of lipid droplets with BODIPY (493/503) (green), nuclei with Hoechst (blue), and anti‐ATGL immunofluorescent staining in red. Scale bar = 50 μm. (B, C) Migration assay. Images were taken at two time points after creating a cell‐free zone. Cells were cultured in the presence of DMSO/atglistatin (left panel) or transfected with siRNA control/siRNA ATGL (right panel). Scale bar = 500 μm. (D, E) Gap closures. Images reported in A, B, and C are representative of *n* = 3 independent experiments. Data are presented in D and E as means ± SD; for D and E, *n* = 3/group. *P < 0.05,***P < 0.001, and ****P < 0.0001 as determined by Student's *t*‐test.

To investigate the impact of ATGL on lipid accumulation and migration of NPC cells, we evaluated lipid load and migration of these cells after inhibition of ATGL by the treatment with atglistatin (an inhibitor of ATGL) or transfection of siRNA ATGL. Lipids in NPC cells were increased upon treatment of atglistatin, although the increase was more dramatic in LMP2A‐negative cells, as expected with their higher expression of ATGL (Fig. 5A left panel and Fig. S1A). ATGL silencing by siRNA also resulted in an increase in lipids, showing a even stronger effect than atglistatin with more and larger lipid droplets in the ATGL‐silenced cells (Fig. 5A right panel and Fig. S1B). Wound‐healing assay was performed to evaluate the migration ability of cells upon atglistatin inhibition or siRNA silencing, followed by BODIPY staining at the time of harvest to investigate a possible correlation between lipid accumulation and gap closure. Measurements of gap width revealed that the inhibition of ATGL by either treatment with atglistatin or siRNA silencing resulted in a faster gap closure than without treatment. This effect was more conspicuous in LMP2A‐negative cells with its higher baseline expression of ATGL (Fig. 5B,C, Hoechst staining). High intracellular lipid load after atglistatin treatment correlated with faster gap closure (Fig. 5B,C, BODIPY staining), and faster migration as shown in Fig. 5D,E.

In addition, the impact of ATGL on lipid levels and cell motility also was evaluated by overexpressing ATGL in LMP2A‐positive and LMP2A‐negative NPC cells. Overexpression of ATGL reduced intracellular lipid droplets (Fig. S2) and slowed down cell migration (Fig. S3).

### 3.6 Downregulation of ATGL correlates with poor overall survival of NPC patients

**Fig. 6:**
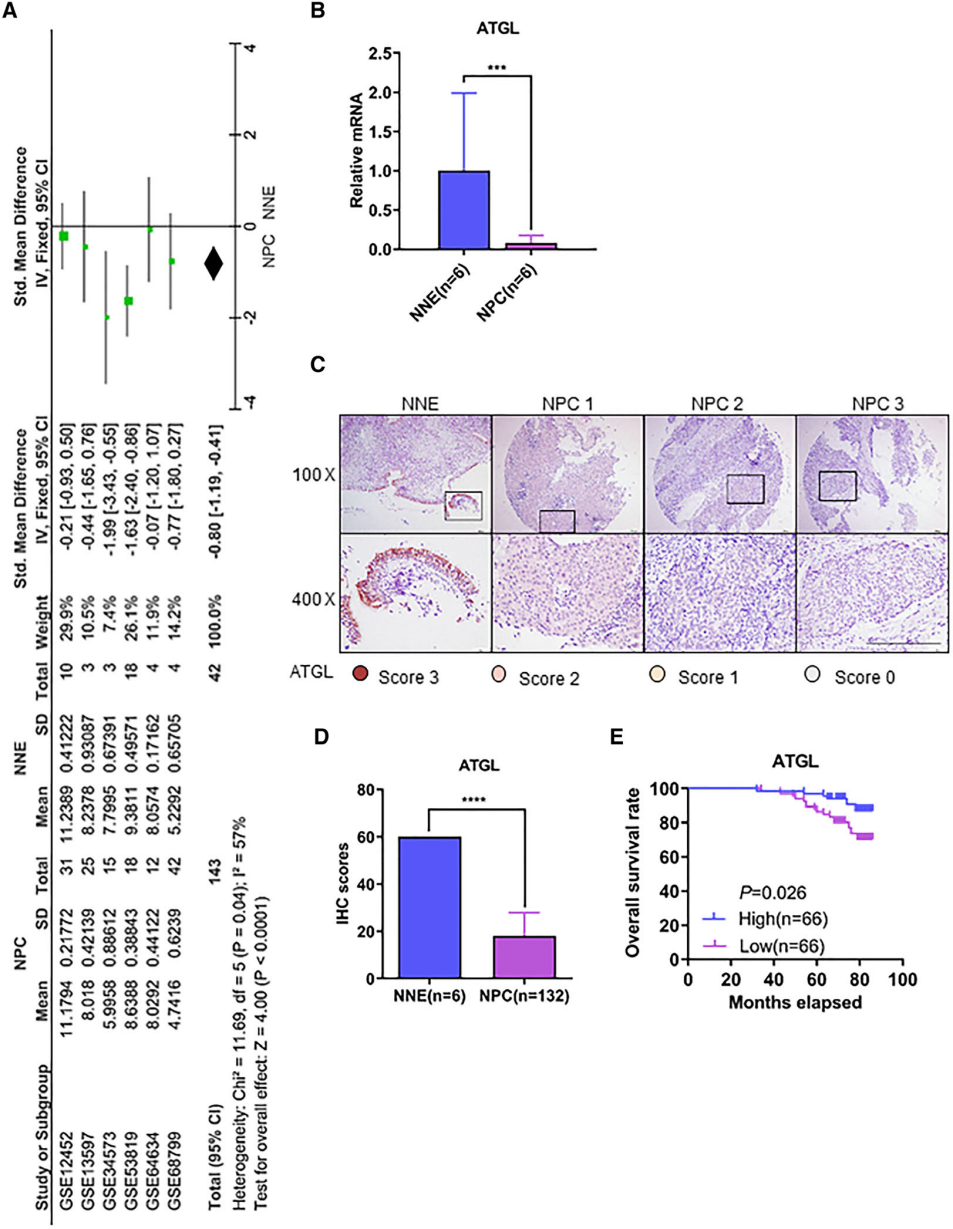
Downregulation of ATGL correlates with poor overall survival of NPC patients. (A) Comparison of ATGL expression in NNE and NPC by meta‐analysis of microarray data. (B) RT–qPCR analysis of relative mRNA level of ATGL in NNE and NPC tissues. (C) Representative IHC staining of ATGL in NNE and NPC cells by a tissue microarray analysis. Scale bar = 200 μm. (D) Immunohistochemistry staining scores for ATGL expression. (E) The overall survival rate of NPC patients with low (*n* = 66) or high (*n* = 66) expression levels of ATGL as estimated by the Kaplan–Meier method for a log‐rank test. The Kaplan–Meier curves were drawn using the graphpad prism 6 software program. ***P < 0.001 and ****P < 0.0001 as determined by Student's *t*‐test.

To test the expression of ATGL in NPC tissues, we compared the mRNA levels of ATGL in samples of NPC tissue and compared this to the normal nasopharyngeal epithelium. A meta‐analysis of human NPC microarrays was performed and revealed that ATGL mRNA was significantly downregulated in NPC tissue (Fig. 6A), which was also validated by qPCR (Fig. 6B). Furthermore, ATGL expression was evaluated by immunohistochemistry staining on tissue microarrays with 132 NPC biopsies. The staining of each biopsy was subjected to semi‐quantitative scoring. Consistent with the qPCR results, the expression of ATGL at the protein level was decreased in NPC biopsies (Fig. 6C,D). To assign IHC scores for ATGL expression, we set the median score as the cutoff value and divided patients into two groups. Patients with higher expression of ATGL (*n* = 66) had better overall survival rate compared to patients with lower levels of ATGL (*n* = 66) (Fig. 6E) in a > 5‐year follow‐up. Thus, the downregulation of ATGL correlated with a worse prognosis for NPC patients.

## 4 Discussion

EBV‐positive NPC cell lines and NPC tumors in southeast of China, which are almost exclusively EBV‐positive and express LMP2A, showed increased levels of lipid droplets [35]. We now explored whether LMP2A expression could explain this change in lipid accumulation, by characterizing the cellular metabolism of LMP2A‐expressing cells.

In contrast to several other tumor viruses, studies of changes in cellular metabolism after EBV infection and in EBV‐associated cancers are limited. It is well established that tumor viruses can induce lipid accumulation in host cells through alternative mechanisms. The mechanism of hepatitis C virus‐induced lipid accumulation has been studied extensively and includes lipogenesis induced by sterol regulatory element‐binding proteins [50], reduction in fatty acids oxidation [51], and disruption of lipoprotein secretion [52]. Viperin and PKR‐like endoplasmic reticulum kinase were reported to induce lipogenesis during human cytomegalovirus infection [53, 54] and Kaposi's sarcoma‐associated herpesvirus infection leads to the increase in the synthesis of long‐chain fatty acids and to increased lipid droplets formation [55]. In NPC, *de novo* fatty acid biosynthesis pathway is also significantly activated. The EBV‐encoded RNAs have been shown to induce this activation via upregulation of FASN, often affected in cancers, and the low‐density lipoprotein receptor [56].

We show that the enhanced lipid accumulation in LMP2A‐positive NPC cells is primarily due to inhibition of lipolysis pathways. An integrative meta‐analysis of NPC transcriptome data from 10 independent NPC microarray datasets, including 135 samples from NPC primary cell lines, cell lines and tissues, identified several deregulated lipid metabolic pathways in NPC, such as the lipid degradation pathway, steroid hormone biosynthesis pathway, and the glycosphingolipid biosynthesis pathway [57]. Our meta‐analysis of microarrays reveals that lipolytic gene ATGL is downregulated in NPC. These findings suggest that downregulation of the lipolytic pathways is an important mechanism for lipid accumulation in EBV‐positive NPC.

As we have seen, LMP2A‐positive NPC cells demonstrate the metabolic shift to aerobic glycolysis. In line with this, LMP2A alters the bioenergetic activity of mitochondria in NPC cells, characterized by decrease in acetyl‐coenzyme A synthesis and oxygen consumption. Many functions of mitochondria have been linked to the antagonistic processes of fission and fusion [58]. It has been reported that LMP2A expression in epithelial cells is associated with increased mitochondrial fission [59] which results in decreased ATP production and generation of reactive oxygen species (ROS) [60]. In this study, we found more ROS in LMP2A‐positive cells (Fig. S4). There is a mutual interaction between the mitochondrial bioenergetic activity and the cytoplasmic metabolism [60], and as a result, either deregulated metabolism or perturbed mitochondrial function can be cause or effect. In our work, we found that LMP2A‐positive NPC cells show decreased bioenergetic activities of mitochondria and lipolysis. The decreased mitochondrial activity results in less acetyl‐coenzyme A available for lipid synthesis. As we show, the enhancement of lipid accumulation in LMP2A‐positive cells is due to inhibition of lipolysis. Due to the decreased lipolysis, LMP2A‐positive cells may not be able to generate enough ATP by fatty acid oxidation. To meet the ATP requirement, they might rather enhance the glycolysis.

We have previously shown that the expression of ATGL is decreased in EBV‐positive NPC cells (C666‐1) via a pathway leading to proteasomal degradation [40]. In the current work, we found that lower expression level of ATGL, a central lipolytic gene, correlated with worse prognosis in NPC patients. Triacylglycerol in the lipid droplets can be fully hydrolyzed to release three fatty acids through the sequential actions of enzymes ATGL, HSL, and MGLL, of which ATGL is the rate‐limiting step [61]. ATGL was reported to be downregulated or lost in several human malignant tumors and low levels of ATGL correlate with significantly reduced survival rate in patients with ovarian, breast, gastric, and non‐small cell lung cancers [62]. ATGL deficiency induces pulmonary neoplasia in an animal model [62], and lung cancer cells depleted of ATGL migrate faster by activation of pro‐oncogenic signaling via SRC kinase and increased levels of bioactive lipids [63]. Overexpression of ATGL promotes glycolytic‐to‐oxidative metabolic switch and cell proliferation in parallel with increased oxidative metabolism of fatty acids and enhanced mitochondrial capacity in hepatocellular carcinoma cells, which reflects ATGL function as tumor suppressor [64]. The role of HSL in cancer is yet unclear. With respect to MGLL, previous studies showed that MGLL expression and activity are elevated in several aggressive cancer cell lines and primary tumors [65, 66]. In contrast to these studies, we did not find changes in HSL and MGLL expression in the LMP2A‐positive NPC cells, but we observed that LMP2A decreased the ATGL expression significantly at both the mRNA level and protein level. In addition, we also found that ATGL downregulation occurs in parallel with enhanced glycolysis and attenuated mitochondrial function in LMP2A‐positive NPC cells. It is well established that LMP2A enhances NPC cell migration. Several mechanisms have been reported, such as via Syk and integrins [31, 67], via EGFR/Ca2+/calpain/ITGβ4 axis[39], or via promotion of epithelial–mesenchymal transition and induction of stem cell characteristics [68]. We now show that LMP2A via downregulation of ATGL increases cell migration *in vitro*. It is possible that this is a common step downstream of several of the reported pathways leading to LMP2A‐enhanced migration.

We mimicked and enhanced downregulation of ATGL by two independent methods, with siRNA knockdown and with an inhibitor atglistatin. The specificity of atglistatin to human ATGL has been controversial [69]. However, our results were very similar to those with siRNA knockdown showing lipid droplet accumulation and increased migration, supporting the specificity of the inhibitor in our human NPC cell line model in line with studies in several other human systems [42, 43, 44, 63, 70, 71].

Interestingly, ATGL is the specific receptor of pigment epithelium‐derived factor (PEDF), which is a tumor suppressor gene [72, 73]. A connection between tumor invasion, PEDF depletion, and lipid accumulation has been established in the pancreas [74] and PEDF also functions as tumor suppressor gene in the process of epithelial–mesenchymal transition and metastasis in NPC [75]. While several studies have shown that PEDF regulates triglycerol dependent on ATGL polyubiquitination [73, 76], so ATGL might function as a hub regulating the lipid metabolism and tumor development.

To summarize, although lipid accumulation by oncoviruses in host cells is usually caused by increase in *de novo* fatty acid biosynthesis, downregulation of lipolysis is an alternative mechanism of lipid accumulation in host cells. Obviously, the intracellular lipid load is determined by the balance between uptake and *de novo* synthesis of lipids on the one hand and lipolysis on the other. LMP2A induces enhanced lipid accumulation and migration in NPC by regulating the key gene ATGL.

## 5 Conclusions

In summary, our work demonstrates a novel mechanism of viral interference with lipid accumulation in NPC. Downregulation of lipolysis in LMP2A‐expressing NPC cells induces enhanced lipid accumulation. ATGL is significantly downregulated in LMP2A‐expressing NPC cells and NPC biopsies, and the reduced expression level of ATGL correlates with poor prognosis in NPC patients.

This may relate to the increased migration induced by LMP2A through ATGL downregulation. These novel findings have implications for a better understanding the regulation of lipid metabolism by viruses and in oncovirus‐associated cancers. Additional studies are required to explore detailed mechanisms of LMP2A‐mediated ATGL downregulation at the protein level, and the relevance of our findings *in vivo* and whether re‐expression of ATGL might affect the malignant phenotype of cancer cells.

## Conflict of interest

The authors declare no conflict of interest.

## Author contributions

IE, LM, and ZZ designed and managed the project. SZ and LM prepared cell samples, extracted RNA, and ran qPCR. ZZ, XX, and XZ prepared tissue samples. SZ and LM contributed to the quantification of intracellular metabolites. LM performed western blotting. SZ and ZZ performed mass spectrometry analysis. SZ, XZ, and ZZ conducted microarray analysis. SZ and LM performed BODIPY staining. SZ and IE analyzed the data of Seahorse experiments. SZ and XX performed IHC staining. SX, GH, and ZZ scored these IHC staining images. SZ, LM, and IE wrote the paper. All authors read and approved the manuscript.

## Supporting information


**Fig. S1.** Inhibition of ATGL by atglistatin or siRNA enhanced lipid accumulation in NPC cells. A. Staining of lipid droplets with BODIPY (493/503) (green), nuclei with Hoechst (blue). B. Anti‐ATGL immunofluorescent staining in red, BODIPY staining of lipid droplets in green and nuclei staining in blue. Scale bar = 200 μm. Images reported in A and B are representative of n = 3 independent experiments.

**Fig. S2.** Overexpression of ATGL reduces lipid accumulation in NPC cells. A and B. Anti‐ATGL immunofluorescent staining in red, BODIPY staining of lipid droplets in green and nuclei staining in blue. Scale bar = 200 μm. Images reported in A and B are representative of n = 3 independent experiments.

**Fig. S3.** Overexpression of ATGL reduces cell migration capacity in NPC cells. A and B. Representative western blot images of ATGL from cells transfected with either td‐Tomato as control or ATGL plasmid DNA. C and D. Migration assay. Images were taken at two time points after creating a cell‐free zone. Scale bar = 500 μm. Images reported in A and B are representative of n = 2 independent experiments.

**Fig. S4.** Increased intracellular reactive oxygen species in LMP2A positive NPC cells. A. Fluorescent DCFH‐DA staining of reactive oxygen species (ROS) (green) Scale bar = 500 μm. B. Analysis of DCFH‐DA staining as determined by fluorescence microplate reader. Images reported in A are representative of n = 3 independent experiments. Data are presented in B as means ± SD; n = 3/group. *P < 0,05 and **P < 0,005 as determined by Student's t‐test.

## Data Availability

The results of microarray analysis were uploaded to Gene Expression Omnibus as GSE118807. The data and materials associated with the current study are available from the corresponding author upon reasonable request.
