# Supplementary figures and images for: Downregulation of adipose triglyceride lipase by EB viral‐encoded LMP2A links lipid accumulation to increased migration in nasopharyngeal carcinoma
Source: Mol Oncol. 2020 Nov 8;14(12):3234–52. doi: 10.1002/1878-0261.12824 (PMC7718958; doi:10.1002/1878-0261.12824)

Supplementary Fig. S1

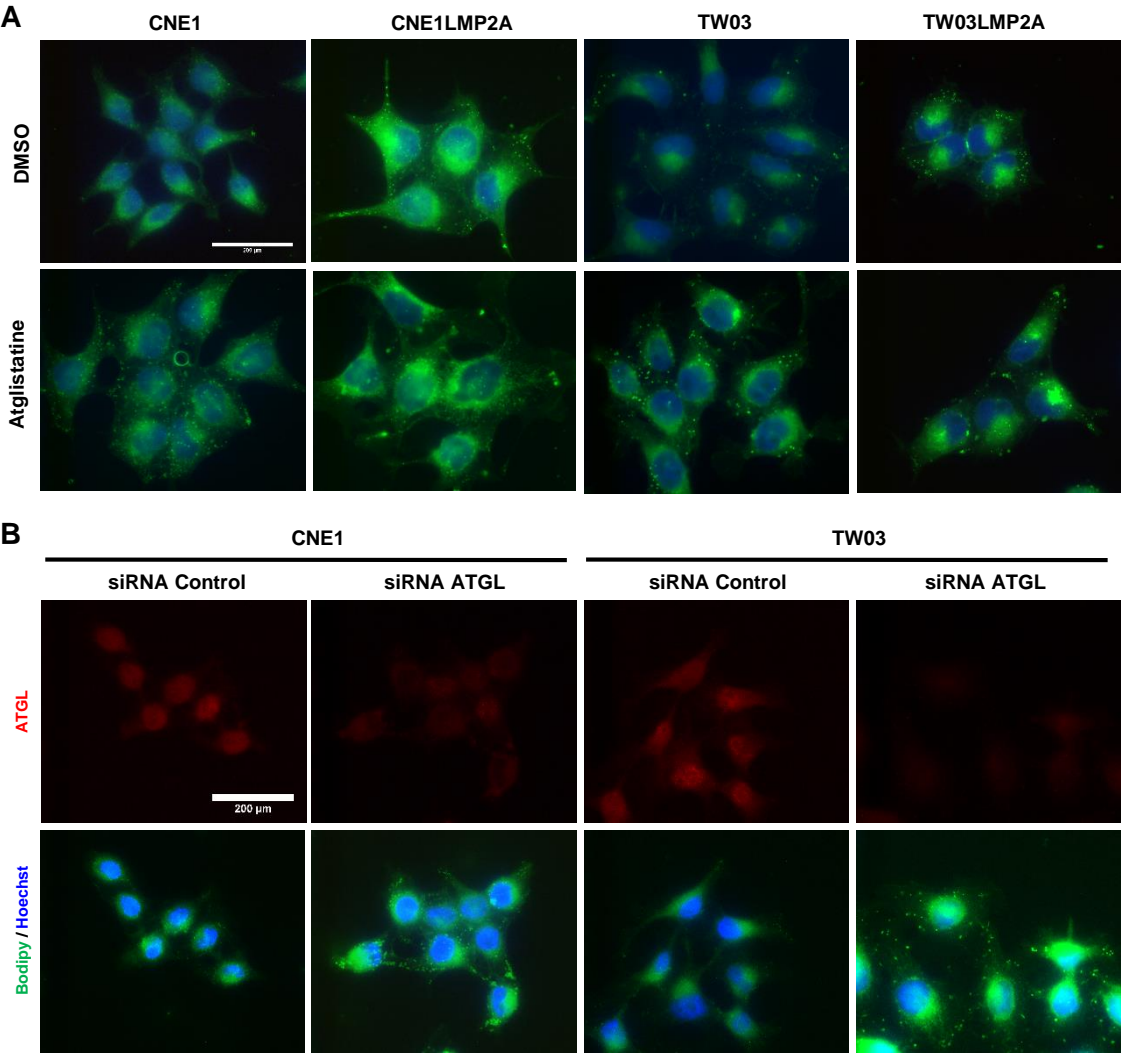

Supplement: Supplementary file 1 — Fig. S1. Inhibition of ATGL by atglistatin or siRNA enhanced lipid accumulation in NPC cells. A. Staining of lipid droplets with BODIPY (493/503) (green), nuclei with Hoechst (blue). B. Anti‐ATGL immunofluorescent staining in red, BODIPY staining of lipid droplets in green and nuclei staining in blue. Scale bar = 200 μm. Images reported in A and B are representative of n = 3 independent experiments. [file MOL2-14-3234-s001.pdf]

Supplementary Fig. S2

A

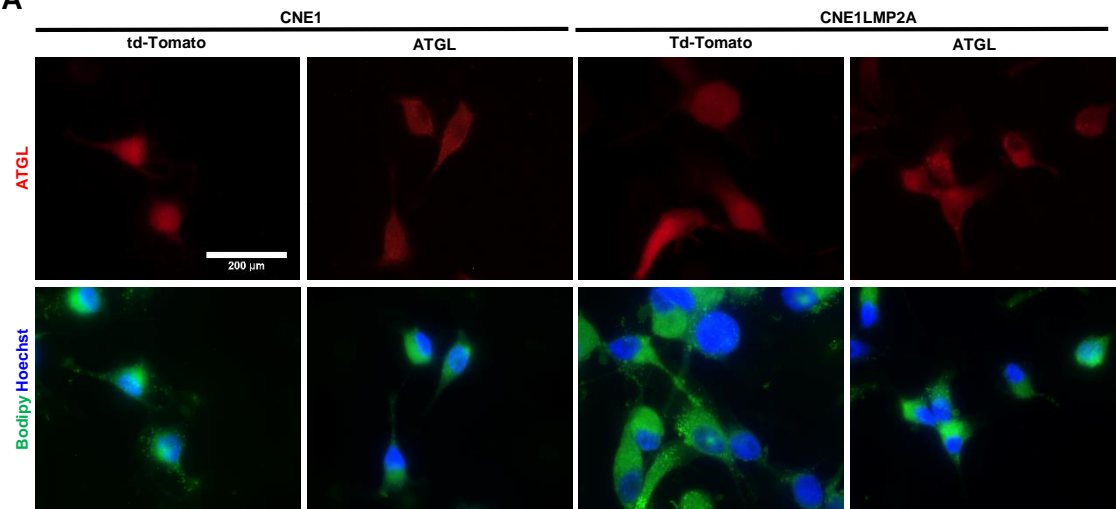

B

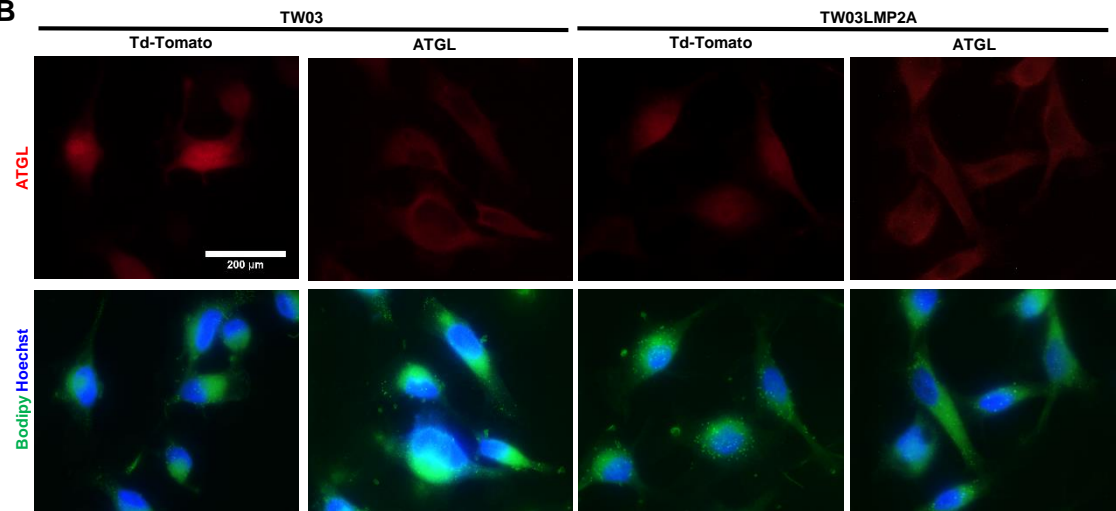

Supplement: Supplementary file 2 — Fig. S2. Overexpression of ATGL reduces lipid accumulation in NPC cells. A and B. Anti‐ATGL immunofluorescent staining in red, BODIPY staining of lipid droplets in green and nuclei staining in blue. Scale bar = 200 μm. Images reported in A and B are representative of n = 3 independent experiments. [file MOL2-14-3234-s002.pdf]

Supplementary Fig. S3

A

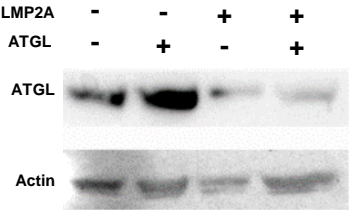

B

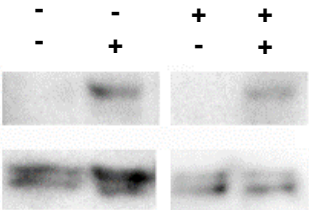

C

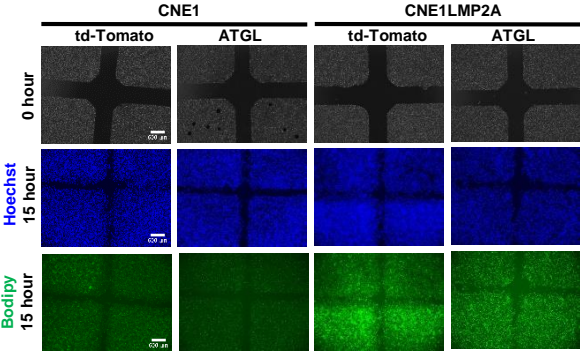

D

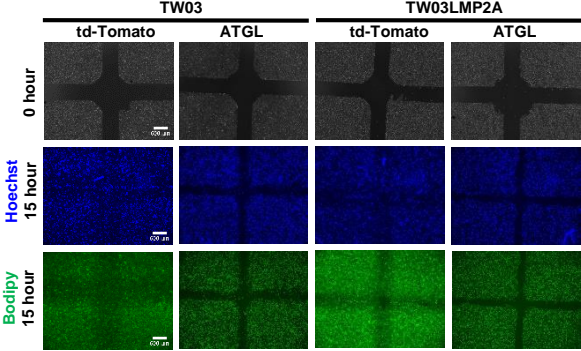

Supplement: Supplementary file 3 — Fig. S3. Overexpression of ATGL reduces cell migration capacity in NPC cells. A and B. Representative western blot images of ATGL from cells transfected with either td‐Tomato as control or ATGL plasmid DNA. C and D. Migration assay. Images were taken at two time points after creating a cell‐free zone. Scale bar = 500 μm. Images reported in A and B are representative of n = 2 independent experiments. [file MOL2-14-3234-s003.pdf]

Supplementary Fig. S4

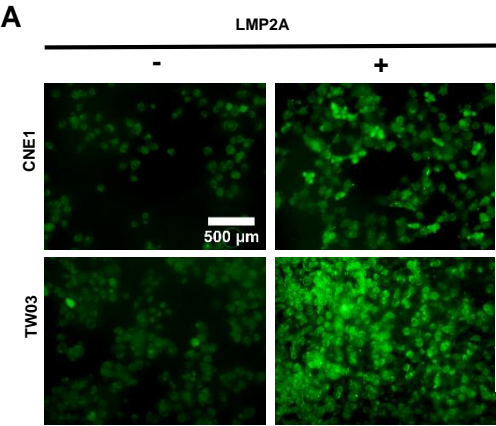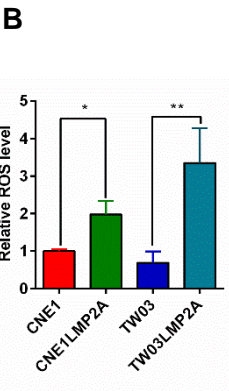

Supplement: Supplementary file 4 — Fig. S4. Increased intracellular reactive oxygen species in LMP2A positive NPC cells. A. Fluorescent DCFH‐DA staining of reactive oxygen species (ROS) (green) Scale bar = 500 μm. B. Analysis of DCFH‐DA staining as determined by fluorescence microplate reader. Images reported in A are representative of n = 3 independent experiments. Data are presented in B as means ± SD; n = 3/group. *P < 0,05 and **P < 0,005 as determined by Student's t‐test. [file MOL2-14-3234-s004.pdf]
